# Rheological Properties of Corn Starch Gels With the Addition of Hydroxypropyl Methylcellulose of Different Viscosities

**DOI:** 10.3389/fnut.2022.866789

**Published:** 2022-03-22

**Authors:** Leticia Montes, Cristina M. Rosell, Ramón Moreira

**Affiliations:** ^1^Department of Chemical Engineering, Universidade de Santiago de Compostela, Santiago de Compostela, Spain; ^2^Institute of Agrochemistry and Food Technology (IATA-CSIC), Paterna, Spain

**Keywords:** average viscosimetric weight, creep and recovery, gelatinization, intrinsic viscosity, viscoelasticity

## Abstract

The objective of this study is to determine the effect of the addition of hydroxypropyl methylcellulose (HPMC) (from 0.5 to 2.0% w/w, starch basis) with three different viscosities (40–60, 80–120, and 2,600–5,600 mPa⋅s) to corn starch (30% w/w, total basis) gels. Average viscosimetric molecular weights (M_*v*_) of tested HPMC were determined (from 27.2 × 10^3^ to 82.7 × 10^3^ g/mol). Water retention capacity of HPMC varied linearly with M_*v*_. The formation and curation of gels were monitored by rheology employing consecutive steps such as heating ramp (25–90°C), time sweep (90°C), cooling ramp (90–25°C), time sweep (25°C), and frequency sweep. Additionally, creep-recovery tests were performed. HPMC above 1.5% w/w delayed the range of gelatinization temperature of starch up to 2°C. Viscoelasticity and stiffness of corn starch gels with HPMC depend on both the amount of polymer added and M_*v*_ of the HPMC. Finally, to achieve corn gels with mimetic viscoelastic properties to wheat gel (with constant total solids), HPMC with relatively low viscosity (low M_*v*_) is necessary to be added at certain content.

## Introduction

Nowadays, gluten-free products are increasingly in demand due to more people are diagnosed as having celiac disease. Most bakery products are made with wheat flour that contains gluten, but there are flours (some of them underexploited) such as corn, rice, chestnut, and acorn, among others, useful to produce gluten-free starchy food materials (1). However, these flours show some poor performance during kneading, proofing, and baking. For this reason, some additives, such as hydrocolloids or gums, are usually included in recipes of gluten-free products to replace the networking properties of gluten.

Hydrocolloids are capable to control the swelling power, solubility, and rheology of starch aqueous systems, throughout the stabilization of emulsions, suspensions, and foams. In fact, they are widely used in the manufacturing of starchy foodstuffs improving the properties of starch gels (2).

A common hydrocolloid used for cited purposes is the hydroxypropyl methylcellulose (HPMC) that belongs to the group of cellulose ethers and has a wide range of applications in food, cosmetics, adhesives, agriculture, and textiles (3). HPMC is hydrophilic, biodegradable, and their solutions exhibit shear-thinning behavior (4). There are many studies that show how the addition of HPMC modifies the viscoelasticity of gluten-free systems to achieve the viscoelastic characteristics of wheat. Zhang et al. (5) studied the effect of adding 2% w/w, starch basis (s.b.) of HPMC (166,700 kDa, and its degrees of methoxyl and hydroxypropyl substitution were 27.2 and 6.8%, respectively) to 5 different starches (wheat, corn, tapioca, sweet potato, and potato) on the rheological properties and found that for each source of starch the interactions between starch and HPMC were predominantly physical linkages and showed a different behavior depending on water-binding capacity of the starch. Similar conclusions were previously obtained with the HPMC addition in corn and potato starches (6). Mancebo et al. (7) studied the influence of HPMC (4,000 mPa⋅s for 2% aqueous solutions at 20°C) (2–4% w/w, by addition, flour basis, f. b.) on rice doughs analyzing the rheological properties and found that elastic modulus (G′) noticeably increased. Sivaramakrishnan et al. (8) studied the addition of HPMC (from 1.5 to 4.5%, w/w, by addition, f. b.) of low viscosity (40–60 mPa⋅s for 2% aqueous solutions at 20°C) to rice flours and, in general, elastic (G′) and viscous (Gȃ) moduli increased as the amount of HPMC increased. Gujral et al. (9) observed that adding 6% w/w, by addition, f. b., of HPMC (4,000 mPa⋅s) to rice flour both moduli increased and, dough consistency increased by 120% increasing HPMC content from 2 to 6% w/w. However, Moreira et al. (10) observed that the incorporation of HPMC (2% w/w, by substitution, f. b.) to chestnut flour decreased G′ and tan δ (G″/G′). Bárcenas et al. (11) used HPMC [from 0.002 to 0.013 g of HPMC/g of wheat starch (WS), by substitution] of high viscosity (4,500 mPa⋅s for 2% aqueous solutions at 20°C) on wheat flour and viscoelastic moduli (G′ and G″) and tan δ decreased with polymer amount. Techawipharat et al. (12) analyzed the effect of adding HPMC (0.8% w/w, by substitution, unspecified viscosity) to rice starch (7.2% w/w) dispersions and observed that both peak viscosity and final viscosity decreased and pasting temperature increased with the HPMC substitution. Moreover, Kim et al. (13) studied the effects of some cellulose derivatives on pea starch. The HPMC used had different viscosities from 45 to 116,640 mPa⋅s (for 2% aqueous solutions at 20°C) and different ratios of methoxyl and hydroxypropyl substitution. Specifically, regarding HPMC with the same substitution degree, the use of high viscosity HPMC increased G′, G″, tan δ, and complex viscosity. Lee et al. (14) studied the effect of adding HPMC of 3 different viscosities (50, 400, and 4,000 mPa⋅s) to waxy rice starch at a ratio of 19:1 (w/w) and the total solids content was 25% w/w. These authors observed that the onset gelatinization temperature increased and the final gelatinization temperature decreased for samples with HPMC. Finally, these authors also found that peak and final viscosity decreased with the HPMC addition except for the sample with the addition of the highest viscosity HPMC. These results clearly indicate that, at constant hydration, the procedure of HPMC adjunction of the starchy system by addition (increasing total solids mass) or by substitution (constant total solids mass) and HPMC viscosity are key characteristics to be considered (15) in the gel characteristics. Globally, focusing on gluten-free products, studies have shown that incorporation of hydrocolloids by addition increased the viscosity and viscoelastic moduli of starch pastes by influencing the gelatinization and retrogradation of starch (3). However, lower moduli and viscosity values were found by the addition of hydrocolloids by substitution.

In relation to the effect of the addition of hydrocolloids with different sizes to corn starch (CS), Funami et al. (16) studied the gelatinization behavior of CS (15% w/w) in the presence of guar gum with different molecular weights (from 0.02 to 34.6 × 10^5^ g/mol) and, at constant guar gum content (0.5% w/w, s. b.) the peak viscosity and setback increased with the lowest molecular weight guar gum and the addition of guar gum delayed the pasting temperature from 75.7°C (for CS) up to 79.6°C.

In these previous studies, the effect of the addition of some hydrocolloids, such as xanthan gum, guar gum, and HPMC, among others, was investigated but without considering the effect of molecular size of HPMC. Likewise, considering the specific interaction depending on the starch source, it was hypothesized that the molecular size of HPMC added to CS, commonly used in gluten-free products, might affect starch interactions and in consequence viscoelasticity of CS gels. Therefore, the aim of this study is to determine the effect of the addition of three different HPMC with different molecular weights on CS gels by means of the evaluation of the viscoelastic behavior.

## Materials and Methods

### Materials

Corn starch [moisture content of 11.4 ± 0.2 dry basis (% d. b.), amylose content 28.1 ± 2.4% d. b.] and wheat starch (moisture content of 12.3 ± 0.2% d. b., amylose content 27.6 ± 0.3% d. b.). HPMC, with constant methoxyl and hydroxypropyl content, 28.7 and 9.1%, respectively, of three different apparent viscosities, 40–60 (HPMC L), 80–120 (HPMC M), and 2,600–5,600 cP (HPMC H), at 2% in H_2_O at 20°C, [moisture content (d. b.) of 4.0 ± 0.1, 3.0 ± 0.2, and 4.9 ± 0.2, respectively]. All the materials were provided by Sigma Aldrich.

### Average Viscosimetric Molecular Weights of Hydroxypropyl Methylcellulose

Average viscosimetric molecular weights were determined by viscosity measurements, using a Ubbelohde type viscometer (AVS 350, Schott-Geräte, GmbH, Germany). Solutions were prepared with a concentration of 0.1% (w/w, d. b.) in distilled water. For each HPMC, three dilutions were performed (0.025, 0.050, and 0.075%). All measurements (at least 5 replicates) were performed at 25°C (± 0.1°C). At these low hydrocolloid content, density was constant and equal to the density of solvent (water). With the absolute viscosities of solvent (μ_0_) and HPMC (μ) solutions, the relative viscosity (μ_μ_ = μ/_0_) and the specific viscosity (μ_*sp*_ = μ_*r*_− 1) were calculated. Using the values of μ_*r*_ and μ_*sp*_, the intrinsic viscosity (μ) was calculated by the Huggins (Eq. 1) and Kraemer (Eq. 2) Equations (17):


(1) $$\mu_{r\,e\,d}=\left[\mu \right]+K_{H}\,{\left[\mu \right]}^{2}\,C$$

(2) $$\mu_{i\,n\,h}=\left[\mu \right]+K_{K}\,{\left[\mu \right]}^{2}\,C$$

where μ_*red*_ = μ_*sp*_/C, μ_*inh*_=*ln*(μ_*r*_)/C, and K_*H*_ and K_*K*_ are the Huggins and Kraemer constants, respectively. The values of viscosity average molecular weight, M_*v*_, were determined by the Mark–Houwink Equation (Eq. 3):


(3) $$\left[\mu \right]=K\,M_{v}^{\alpha }\;$$

where (μ) (dl/g) is the intrinsic viscosity and K and α values depended on the solute–solvent system at a constant temperature.

### Water Retention Capacity

Water retention capacity (WRC) values were determined to characterize the CS and HPMC-CS mixtures. WRC was calculated following the protocol established by Robertson et al. (18). Briefly, samples were weighed and hydrated for 18 h. After the samples were centrifuged, the supernatant was removed and the solid residue was dried to constant weight (dry residue). The WRC (kg^–1^) was calculated by Eq. (4).


(4) $$W\,R\,C=\frac{F\,R\,W-D\,R\,W}{D\,R\,W}$$

where *FRW* is the fresh residue weight and *DRW* is the dry residue weight. WRC of HPMC was not directly determined due to water forms a viscous gel (19), but it was indirectly evaluated by means of HPMC and CS mixtures. WRC_*HPMC*_ was evaluated by the Eq. (5):


(5) $$W\,R\,C_{H\,P\,M\,C}=\frac{W\,R\,C_{m\,i\,x\,t\,u\,r\,e}-W\,R\,C_{C\,S}\,\left(1-\frac{w_{d\,H\,P\,M\,C}}{\left(w_{d\,C\,S}+w_{d\,H\,P\,M\,C}\right)}\right)}{\frac{w_{d\,H\,P\,M\,C}}{\left(w_{d\,C\,S}+w_{d\,H\,P\,M\,C}\right)}}\;$$

where *WRC*_*CS*_ is the WRC of CS, *w*_*dHPMC*_ and *w*_*dCS*_ are the dry weights of HPMC and starch in the mixture, respectively.

### Rheological Properties

Dispersions with constant solids content (30% w/w) were prepared by mixing CS and HPMC (by substitution) at different concentrations (0, 0.5, 1.0, 1.5, and 2.0% w/w) and distilled water. First, the solids were gently blended and later distilled water was added. The slurry was mildly stirred (100 rpm) using a magnetic stirrer, for 10 min at room temperature. Aqueous HPMC solutions (2.0% w/w) were prepared by mixing HPMC and distilled water. These mixtures were kept in agitation until completely dissolved. The rheological characterization was performed with a stress-controlled rheometer (MCR 301; Anton Paar Physica, Graz, Austria) using a plate–plate geometry (diameter, 50 mm). Samples (1.2 ml) were loaded between the parallel plates and compressed up to obtain a gap of 0.5 mm. In the case of HPMC solutions, the same geometry was used with a smaller gap (0.3 mm) with a volume of sample of 0.9 ml. The measurements were performed at different temperatures [from 25 up to 90°C (± 0.1°C)], controlled by a Peltier system. All the samples were covered with light paraffin oil to prevent water evaporation. Tests were carried out at least in triplicate.

#### Oscillatory Measurements

**FIGURE 1:**
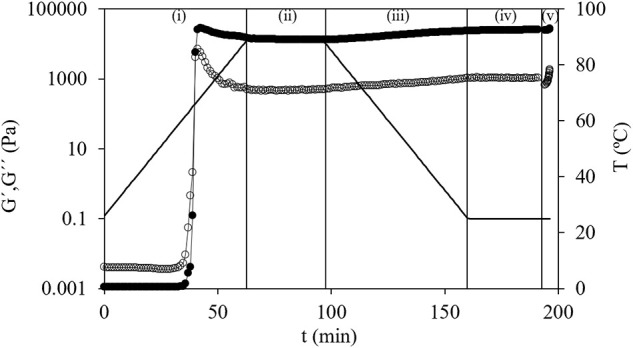
The general outline of the five steps of oscillatory measurements of G′(●) and G″(○) of corn starch and temperature (–) vs. time: (i) temperature sweep (25–90°C at 1°C min^–1^, 1 Hz, and 100 Pa); (ii) time sweep (30 min, 1 Hz, 400 Pa, and 90°C); (iii) temperature sweep (90–25°C at 1°C min^–1^, 1 Hz, and 400 Pa); (iv) time sweep (30 min, 1 Hz, 400 Pa, and 25°C); and (v) frequency sweep (0.01–100 Hz, 1% of strain, and 25°C).

Strain sweep tests from 0.1 to 10% at a constant frequency (1 Hz) were made on dispersions and gels to define the corresponding linear viscoelastic regions (LVRs). Second, dispersions were subjected to the procedure previously reported (20) with minor modifications consisting in five steps: (i) temperature sweep (25–90°C at 1°C min^–1^, 1 Hz, 100 Pa), to accomplish starch gelatinization; (ii) time sweep (30 min, 1 Hz, 400 Pa, 90°C); (iii) temperature sweep (90–25°C at 1°C min^–1^, 1 Hz, 400 Pa); (iv) time sweep (30 min, 1 Hz, 400 Pa, 25°C); and finally (v) frequency sweep (0.01–100 Hz, 1% of strain, 25°C) inside LVR of formed gels (preliminary tests were performed to determine the corresponding LVR). For HPMC solutions (2.0% w/w in water) a strain sweep from 0.1 to 100% at a constant frequency (1 Hz) was made to analyze the LVR. Then, a temperature sweep was carried out from 35 to 80°C at a constant strain of 10% and a constant frequency of 1 Hz (inside LVR). Figure 1 shows a general outline of these five steps where viscoelastic properties were evaluated over time.

#### Creep-Recovery Test

A creep-recovery test at 25°C was performed at constant stress, σ (Pa), of 100 or 400 Pa within the LVR during the creep phase. Before the measurements, the gels were rested for 15 min in the rheometer to allow the sample equilibrium. During the creep period, the selected constant stress was applied for 120 s and the recovery (stress zero) period lasted 120 s (21). The results were analyzed in terms of creep compliance, J(t) (Pa^–1^) = γ/σ, where γ is the strain experimentally measured. The Burgers model (22) was employed for the creep (Eq. 6) and the recovery phases modeling (Eq. 7):


(6) $$J\left(t\right)=J_{0}+J_{m}(1-\left(\exp\left(-\frac{t}{\lambda }\right)\right)+t/\eta_{0}$$

(7) $$J\,\left(t\right)=J_{m\,a\,x}-J_{0}-J_{m}\,\left(1-\left(\exp\left(-\frac{t}{\lambda }\right)\right)\right)$$

where *J_0_* (Pa^–1^) is the instantaneous compliance, *J_m_* (Pa^–1^) is the viscoelastic compliance, λ (s) is the mean retardation time, *t* (s) is the phase time, η_0_ (Pa⋅s) is the zero-shear viscosity, and *J*_*max*_ (Pa^–1^) is the maximum creep compliance. The goodness of fitting was evaluated by means of the corresponding coefficients of determination [*R*^2^ and the root mean square error (RMSE)].

### Statistical Analysis

Experimental data were analyzed through one-factor analysis of variance (ANOVA), followed by the Duncan test and considering significant *p*-values ≤ 0.05 (IBM SPSS Statistics. New York, NY, United States). All the experimental results were expressed as mean ± SD from at least triplicate experiments.

## Results

### Average Viscosimetric Molecular Weights of Hydroxypropyl Methylcellulose

Table 1 shows the K_*H*_ and K_*K*_ constants, intrinsic viscosity (μ) values, and the average viscosimetric molecular weight (M_*v*_) for each HPMC. M_*v*_ was calculated by the Mark–Houwink equation, Eq. (3), where the values of K and α parameters were 3.39 × 10^–4^ dl/g and 0.88, respectively, previously determined by Vázquez et al. (23). Intrinsic viscosity is a measure of the hydrodynamic volume occupied by the individual polymer molecules in isolation (24). The (μ) and M_*v*_ values varied from 2.72 to 7.21 dl/g and from 27.2 × 10^3^ to 82.7 × 10^3^ g/mol, respectively. Vázquez et al. (23) studied the (μ) values of HPMC with different nominal viscosity (from 10^2^ to 10^5^ mPa⋅s) and obtained values from 3.78 to 14.98 dl/g and M_*v*_ values from 39.7 × 10^3^ to 190.0 × 10^3^ g/mol. Bustamante et al. (25) studied the (μ) of HPMC with 28–30% methoxyl and 7–12% hydroxypropyl content and approximate molecular weight of 86 kDa and obtained a value of 8.09 dl/g. These results agree with the results obtained in this study.

**TABLE 1:** Parameters obtained from the Eqs (1) to (3).

HPMC	Nominal viscosity (mPa⋅s)	K_H_	K_HK_	(μ) (dL/g)	M_Hv_ (10^3^g/mol)
L	40–60	0.21 ± 0.11^a^	−0.26 ± 0.08^a^	2.72 ± 0.15^a^	27.2 ± 1.8^a^
M	80–120	0.47 ± 0.09^b^	−0.08 ± 0.06^b^	3.18 ± 0.08^b^	32.7 ± 1.0^b^
H	2,600–5,600	0.63 ± 0.03^b^	−0.04 ± 0.01^b^	7.21 ± 0.14^c^	82.7 ± 1.8^c^

*Data are presented as mean ± SD. Data value of each parameter with different superscript letters are significantly different (p ≤ 0.05).*

The values of K_*H*_ and K_*K*_ obtained were from 0.21 to 0.63 and from –0.26 to –0.04, respectively. As the nominal viscosity of the HPMC increased, the K_*H*_, K_*K*_, (μ), and M_*v*_ significantly (*p* < 0.05) increased as well. HPMC solubility can be related to intrinsic viscosity (25). This behavior shows that an increase in viscosity (and, therefore, an increase in M_*v*_) causes a decrease in the solubility of the biopolymer in water. The values of K_*H*_ and K_*K*_ could be analyzed to study the interactions between the polymers and solvents. In fact, K_*H*_ < 0.5 and K_*K*_ < 0 indicate good solvents (strong interactions polymer–solvent). An additional criterium given by K_*H*_ – K_*K*_ close to 0.5 based on the solvent goodness (solvent θ), defined a condition in which neither inter- or intramolecular polymer aggregation is produced and particulates behave as non-perturbed units and polymers adopt an extended conformation (flexible coil) in the solvent used (26). Our results indicated that only HPMC H slightly diverged (K_*H*_ = 0.63 > 0.5 and K_*H*_ – K_*K*_ close to 0.7) from theoretical considerations for good solvent–polymer interactions and this fact could be tested out in its low water solubility and slow solubilization.

### Water Retention Capacity

In order to calculate the WRC of the different HPMC samples, mixtures of starch and HPMC were made. By means of the Eq. (4) the WRC of CS and mixtures were calculated. Then, using Eq. (5) the WRC of HPMC was evaluated. WRC_*CS*_ was 0.95 ± 0.01 (g water/g d.b.) whereas WRC values of HPMC L, M, and H, were 8.54 ± 0.28, 8.92 ± 0.22, and 12.97 ± 0.45 (g water/g d.b.), respectively. Yılmaz et al. (27) reported the same effect with the addition of HPMC to wheat-rice and wheat-corn flours. The WRC increased in the samples where HPMC was added. Usually, the hydrophilicity depends on the HPMC substitution degree (28). In this case, all samples had a constant substitution degree, therefore, WRC increased with increasing the M_*v*_ of HPMC meaning that a high molecular weight of polymer implied high water retention. This fact can be explained because in larger polymers, the hydrophilic groups (hydroxypropyl) are more accessible for water and therefore, more water amount could be retained. Additionally, a linear correlation was found between intrinsic viscosity (μ), of HPMC and WRC_*HPMC*_ (*R*^2^ > 0.99). As (μ) was proportional to M_*v*_, WRC_*HPMC*_ also varied linearly with M_*v*_ of HPMC (*R*^2^ > 0.99).

### Rheological Characterization

#### Oscillatory Measurements

**FIGURE 2:**
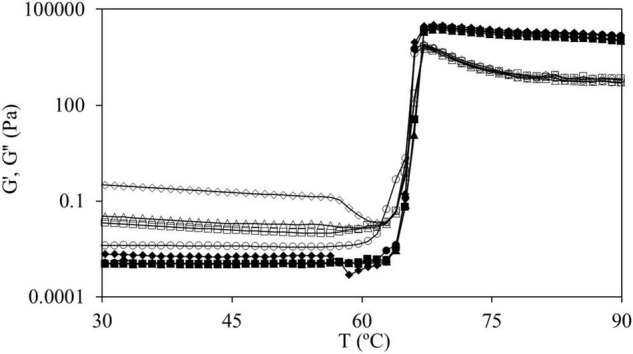
G′ (fully symbols) and G″ (empty symbols) values for temperature sweep (from 30 to 90°C at 1°C min^–1^, 1 Hz, and 100 Pa) for CS (●) and mixtures CS + 2.0% hydroxypropyl methylcellulose (HPMC) for HPMC L (■), HPMC M (▲), and HPMC H (◆).

Dispersions were subjected to a temperature sweep (step i) where the onset gelatinization temperature (T_0_) was evaluated from the first inflection point of elastic modulus (G′) and the final gelatinization temperature (T_*f*_) was determined from the point in which the slope of G′ changes after the peak (29). Figure 2 shows the curves obtained during the temperature sweep for samples of CS and CS + 2.0% HPMC (L, M, and H). During this stage, G′ values drastically increased 7 decades, approximately, and at the end, G′ > G″. From this point, G′ values remained above G″ throughout the experiment, which means that gels have a solid elastic-like behavior. T_0_ obtained for CS gel was 60.7 ± 1.1°C and for gels with HPMC L varied from 61.8 ± 1.1 (for 0.5, 1.0, and 1.5%) to 62.8 ± 1.1°C (for 2.0%), while with HPMC M was 61.8 ± 1.1°C (for 0.5 and 1%) and 62.8 ± 1.1°C (for 1.5 and 2.0%). However, T_0_ value for HPMC H hardly changed, 62.8 ± 0.8°C, independently of its content. In addition, the T_*f*_ was delayed in a similar way, from 74.2 ± 0.8°C for CS gels to 75.3 and 76.4 ± 0.8°C with the presence of HPMC. This delay of the gelatinization process could be related to the water absorption of the added hydrocolloid (30) that competes with starch for the available water. In fact, the WRC results showed that mixtures of starch with HPMC retained more water than CS without HPMC sample. The same behavior was shown by Moreira et al. (31) in chestnut flour doughs with the addition of 0.5–2% HPMC (viscosity 2,600–5,600 cP, 2% in H_2_O at 20°C) by substitution. Moreover, Zhang et al. (32) showed the same trend with other hydrocolloids such as arabic gum, guar gum, and xanthan gum added by substitution. Also, Alamri et al. (33) reported that okra extract delayed the onset temperature in WS.

**FIGURE 3:**
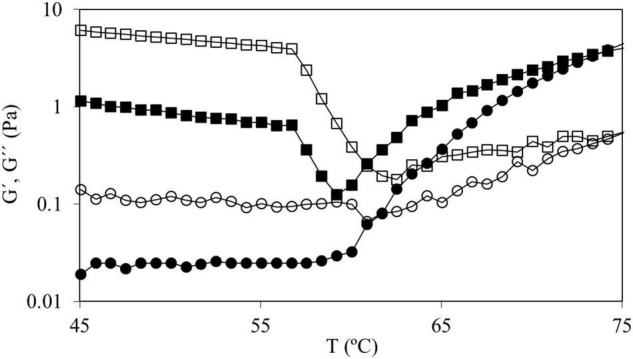
Temperature sweeps from 45 to 75°C of HPMC solutions 2.0% w/w in water for HPMC L (●) and HPMC H (■) where G′ has full symbols and G″ has empty symbols.

In the curves of CS and in those of CS + 2.0%, HPMC L and HPMC M both moduli remained constant until reaching T_0_ (onset starch gelatinization temperature) (Figure 2). For the mixtures of CS with HPMC (M and L), the same behavior was observed at all the HPMC content studied (data not shown). However, for CS + 2.0% HPMC H samples, as the temperature increased, G′ and G″ gently decreased until accomplishing a characteristic temperature (∼57°C) after which a strong decrease is observed. This fact must be related to the thermal behavior of HPMC. To characterize the thermogelation features of HPMC, 2.0% w/w HPMC aqueous solutions were subjected to temperature sweeps (Figure 3).

As it can be observed in Figure 3, in the HPMC L (and HPMC M, data not shown) solution at temperatures below 60°C, both moduli are almost constant (with G″ > G′) and from this temperature, both moduli started to increase (particularly G′) achieving a gel point (G″ = G′) at 61.0 ± 0.8°C and at higher temperatures G′ sharply increased. However, HPMC H solution showed a different trend, a notorious drop (above 57°C) of both moduli was observed before the gel point achievement. Silva et al. (34) observed this effect (using high viscosity HPMC, 15,000 mPa⋅s) and concluded that this process is due to the higher hydrophobicity of the polymer chains promoted during heating, suggesting the existence of strong aggregation phenomena. Temperature sweeps seemed to indicate that this fact could be also promoted by the molecular weight of the polymer due to the described behavior before gel point was only observed with tested high molecular weight HPMC. The temperature of the gel point (around 61°C) was the same for all tested HPMC polymers. HPMC gel point depends on methoxyl and hydroxypropyl substitution degree (35) and our results indicate that it is independent of the average molecular weight of HPMC.

Analyzing in detail the results shown in Figure 2, it can be observed that CS with HPMC H showed the same drop of both the moduli at the mentioned temperature, so in the aqueous dispersion of starch and HPMC, the interactions between polymers before starch gelatinization are negligible and rheological properties are governed by the individual relationships (affinities) of polymers with water.

A time sweep at 90°C for 30 min (step ii) (Figure 1) to evaluate the gel formation rate and to determine the elapsed time until achieve a fully formed gel was carried out. Evaluating G′, around 20 min are necessary to reach a constant value in the case of CS gels. However, the HPMC addition substantially shortened the necessary time, around 5 min, to obtain a stable gel. In parallel, G″ values not varied during this time sweep. The fact that corn gels with HPMC needed short times to get the stability could be an industrial advantage by saving time and costs. Moreover, this fact showed that the HPMC gelation promoted and modified the formation of CS gel. During time sweep, the variations of G′ for tested gels, ΔG_90_′ = G′_90f_ – G′_90i_, were evaluated where G′_90f_ is the G′ after 30 min and G′_90i_ is the initial value. For CS gel, without the addition of HPMC, ΔG_90_′ was −970 Pa, starch gels with HMPC L and M showed lower changes, but always G′_90f_ < G′_90i_. These variations decreased with the amount of HPMC added. However, with the addition of HPMC H, two different trends were observed depending on the amount of polymer used. Negative values of ΔG_90_′ were also determined at low content (0.5% w/w), but increasing HPMC H content (>1.0% w/w) positive ΔG_90_′ values were determined achieving 1,560 Pa at 2.0% w/w. These results showed that M_*v*_ of HPMC clearly modifies the formation rate of gels and their final characteristics. In our tests, despite tested polymers had the same substitution degree, when they were added at the same content, the gel features were significantly different. The addition of HPMC, independently of M_*v*_, dampened the decay of G′ observed in CS gels due to the presence of hydroxyl groups that are capable to form additional hydrogen bonds and therefore stronger structures (36). The positive ΔG_90_′ values found with HPMC H indicated that strengthened gels were formed, confirming that in large polymer molecules the accessibility to the functional groups is facilitated.

In the next stage, the temperature sweep from 90 to 25°C (step iii), Figure 1, it was observed that both the moduli gradually increased with decreasing temperature, corresponding to starch retrogradation (37). Afterward, a maturation period (step iv) of 30 min, time sweep at 25°C, stress (400 Pa), and frequency (1 Hz), was carried out to evaluate gel stability at room temperature. The evolution of G′ with time during this stage indicated that stationary G′ values were obtained at a short time (less than 2.5 min). In all cases, the presence of HPMC decreased the elapsed time to achieve gel stability until below 1 min for HPMC L at 2.0% w/w. A similar analysis performed in time sweep at 90°C was carried out in this stage by evaluation of ΔG_25_′ = G′_25f_ – G′_25i_. Table 2 shows the G′_25i_ and ΔG_25_′ values for tested gels. Globally, G′_25i_ values decreased with increasing polymer addition of HPMC L and M. This result indicated that short-time starch retrogradation given by the rapid recrystallization of amylose molecules was slowed down by these HPMC additions due to the increase of G′ during cooling (step iii) was significantly lower. However, this decrease of G′_25i_ only was observed at the lowest content of HPMC H (0.5% w/w) because beyond that level G′_25i_ showed a steady increase with the level of HPMC addition (2.0% w/w) maintaining the trend observed at 90°C. In fact, the addition of hydrocolloids can increase, decrease, or have no effect on the extent of (short- and long-time) starch retrogradation, depending on the gel preparation method, temperature, time, added amount of hydrocolloid, and measurement techniques of starch retrogradation (38). The aforementioned effect of HPMC on starch retrogradation was confirmed by the corresponding analysis of the time sweeps at 25°C where positive ΔG_25_′ values were obtained, meaning that in all cases G′_25f_ > G′_25i_, Table 2. Nevertheless, the maximum firming up was observed in CS gels (1,420 Pa) with HPMC H at 2.0 w/w (1,460 Pa). HPMC addition diminished ΔG_25_′, but different trends with polymer content were found. At higher HPMC L and M content, ΔG_25_′ values significantly (*p* < 0.05) decreased (up to 865 Pa with HPMC L at 2.0% w/w). No significant differences among ΔG_25_′ values of gels with different amounts of HPMC H added were observed, and these changes were close to that determined in CS gel (*p* > 0.05).

**TABLE 2:** Data from maturation stage obtained at 400 Pa, 1 Hz, and 25°C during 30 min.

Sample	Polymer dosage (% w/w)	G′_25i_ (Pa⋅s)	Δ G_25_′ (Pa⋅s)
CS	0	23,955 ± 431^ef^	1,420 ± 14^fg^
HPMC L	0.5	21,535 ± 1039^c^	1,270 ± 42^d^
	1.0	19,880 ± 283^b^	1,190 ± 38^c^
	1.5	19,630 ± 724^b^	1,010 ± 33^b^
	2.0	17,490 ± 919^a^	865 ± 37^a^
HPMC M	0.5	21,900 ± 212^cd^	1,330 ± 44^de^
	1.0	21,720 ± 397^c^	1,280 ± 41^d^
	1.5	19,155 ± 1308^b^	1,125 ± 64^c^
	2.0	17,450 ± 933^a^	955 ± 35^b^
HPMC H	0.5	22,430 ± 14^cde^	1,335 ± 7^de^
	1.0	23,395 ± 587^def^	1,275 ± 7^d^
	1.5	24,750 ± 339^f^	1,360 ± 12^ef^
	2.0	27,250 ± 120^g^	1,460 ± 15^g^

*Data are presented as mean ± SD. Data values in a column with different superscript letters are significantly different at the p ≤ 0.05 level.*

**FIGURE 4:**
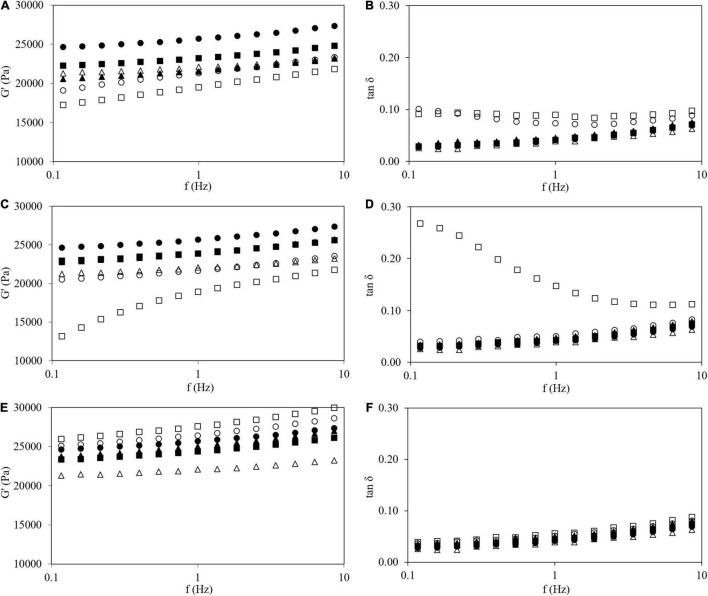
Frequency sweep tests (at 1% of strain, 25°C) of G′ **(A,C,E)** and tan δ **(B,D,F)** of corn starch gel (●), wheat starch (Δ), and corn starch with HPMC L **(A,B)**, M **(C,D)**, and H **(E,F)** gels: 0.5 (●), 1.0 (■), 1,5 (▲), and 2.0% (□).

The previous time sweep (step iv) showed that the gels have reached a stable state and are ready to analyze their structures. For this reason, frequency sweeps (step v) (at constant strain 1%, inside LVR) were carried out to characterize the viscoelastic properties of gels (Figure 4). For all samples, G′ increased with increasing frequency, indicating that samples with or without HPMC are typical weak gels (39, 40). In general, the HPMC L and M addition decreased G′ values. However, the opposite effect was observed when HPMC H was added above 1.0% w/w content. The tan δ (G″/G′) values lower than one indicates a predominance of elastic over viscous properties (5), Figure 4. At high concentrations, the addition of HPMC L and M showed higher values of tan δ, particularly at low frequencies. However, both moduli varied proportionally with HPMC H addition and the damping factor was invariant inside the studied frequency range. Lee et al. (14) observed the same trends employing waxy rice starch with the addition of HPMC of different viscosities with a ratio of 19:1 (w/w) and a total solids content of 25% w/w. Low viscosity HPMC (50 mPa⋅s) addition decreased G′ values in the mechanical spectrum while the addition of high viscosity HPMC (4,000 mPa⋅s) produced the opposite behavior.

For comparative purposes, the frequency sweep for a WS gel (also at 30% w/w) formed under the same conditions that tested CS gels included in Figure 4. Viscoelastic properties of WS gel nicely agreed with data of wheat gel at the same starch content reported by Sasaki et al. (41). WS gel showed lower G′ and similar tan δ than CS gel. Nevertheless, viscoelastic parameters of WS gel could be obtained with the addition of HPMC L (at 1.0% w/w, Figures 4A,B) and HPMC M (at 1.5% w/w, Figures 4C,D). Nevertheless, the use of HPMC H was not useful to reproduce the viscoelastic features of CS gels because despite the adequate damping factor, the G′ values are systematically high, independently of the amount added (Figures 4E,F). Sivaramakrishnan et al. (8) also found that by adding a specific amount of low viscosity HPMC (around 3% w/w) to rice flour, similar viscoelastic characteristics to wheat flour could be obtained.

**FIGURE 5:**
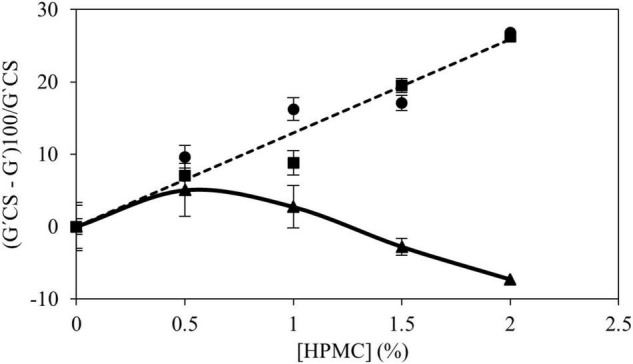
Relationship between the (G′_*CS*_ – G′)100/G′_*CS*_ values obtained from the mechanical spectrum at 1 Hz and the concentration of HPMC: L (●), M (■), and H (▲).

To establish some relationships between the rheological properties of fully developed CS gels with HPMC, G′ from frequency sweeps at 25°C were analyzed. Figure 5 shows the percentage of change of G′ by HPMC addition with respect to the CS gels with values measured at 1 Hz. For gels containing HPMC L and M (low and medium M_*v*_) a positive linear trend was found (*R*^2^ > 0.98) with HPMC content. The firmness of gels with HPMC L and M decreased linearly with the amount of polymer added and no significant differences (*p* > 0.05) between both polymers were found. In samples with HPMC H, it was observed that the previous trend is maintained only at the low addition (0.5% w/w) level. At higher content (>1.0% w/w), the opposite effect is observed, and consequently, the change of G′ diminished (negative values meant that G′ values of gels with HPMC were higher than G′ of CS gels) due to the increase of G′ with additional amounts of polymer added. This fact is given by the high viscosity of HPMC in the continuous phase (14).

#### Creep-Recovery Test

**FIGURE 6:**
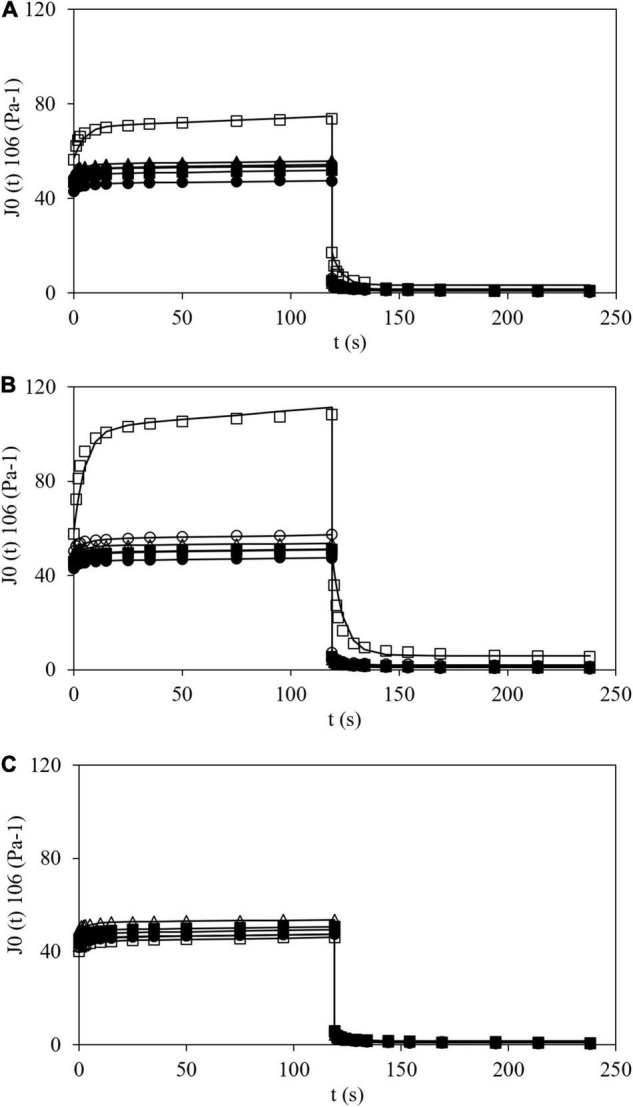
Creep (120 s) and recovery (from 120 to 240 s) plots of corn starch (●), wheat starch (Δ), and corn starch + HPMC L **(A)**, M **(B)**, and H **(C)** at 0.5 (■), 1.0 (▲), 1,5 (○), and 2.0% (□), and modeling (–).

Figure 6 shows creep-recovery curves at 25°C of all the tested samples where typical viscoelastic behavior can be observed. During creep step (120 s) the stress (100 or 400 Pa) was applied and creep compliance, J, was measured. Then, stress was removed, and J was also measured during 120 s (recovery step). For HPMC L and M a similar behavior was shown. In fact, at constant time, during the creep phase, J values increased with HPMC addition. In the case of CS with HPMC H, two different trends were observed. At low addition (0.5% w/w) an increase in J (like the effect observed with HPMC L and H) was displayed, but at higher polymer content (>1.0% w/w) smaller J values than that obtained for CS gels were determined. This is consistent with data obtained and already discussed previously with the oscillatory tests. Additionally, similar curves were obtained by Korus et al. (42) for CS samples.

The Burgers model satisfactorily fitted the experimental creep and recovery data (*R*^2^ > 0.95 and RMSE < 4.8 10^–6^ Pa^–1^). Parameters obtained using the Burgers model (Eqs 6 and 7) are shown in Table 3. The J_0_ values varied from 43.0 10^–6^ Pa^–1^ for CS gel up to 56.5, 57.7, and 40.2 10^–6^ Pa^–1^ for gels at maximum polymer addition (2.0% w/w) of HPMC L, M and H, respectively. For WS gel, J_0_ was 49.6 × 10^–6^ Pa^–1^. The J_*m*_ values obtained varied in a restricted range between 3.3 and 5.3 × 10^–6^ Pa^–1^, excepting for CS gels with HPMC L and M (at 2.0% w/w) which were noticeably higher, 13.92 and 44.70 × 10^–6^ Pa^–1^, respectively. In general, λ and μ_0_ varied between 4.1 and 5.7 s and 286 and 597 Pa⋅s, respectively. The J_*max*_ values varied from 46.2 to 56.1 × 10^–6^ Pa^–1^, excepting for CS gels with HPMC L and M (at 2.0% w/w), 73.6 and 108.4 × 10^–6^ Pa^–1^, respectively. These results were also in agreement with the obtained results by oscillatory tests where G′ and tan δ values varied sharply showing lower G′ values and higher tan δ than remaining samples. J_0_ varied inversely with respect to G′ (from frequency sweep at 0.1 Hz and 25°C) and a successful linear correlation between both the parameters could be established (*R*^2^ > 0.96). Consequently, the trends found for G′ with HPMC amount in the oscillatory tests are also valid here. Similarly, Onyango et al. (43) added HPMC (viscosity not specified) to gluten-free dough at two different concentrations (0.4 and 2.4% w/w f.b.) and observed that with the lowest concentration, J_0_ and J_*m*_ increased and, with the highest concentration, J_0_ and J_*m*_ decreased. J_0_ value can be used to analyze the sample rigidity. In general, HPMC addition increased J_0_ values and, therefore, decreased the gel stiffness. However, the addition of HPMC H causes a different behavior from the threshold concentration, as previously observed. Moreira et al. (44) observed that the creep compliance (J_0_, J_*m*,_ and J_*max*_) values increased when the HPMC content increased in gluten-free doughs based on chestnut flour.

**TABLE 3:** Parameters of Burgers model (Eqs. 6 and 7) for creep and recovery tests.

Sample	HPMC (%w/w)	J_0_ 10^6^ (Pa^–1^)	J_*m*_ 10^6^ (Pa^–1^)	λ (s)	μ _0_ (Pa⋅s)	J_*max*_ 10^6^ (Pa^–1^)	*R* ^2^	RMSE 10^6^ (Pa^–1^)
WS	0	49.6 ± 0.7^b^	3.1 ± 0.1^j^	5.5 ± 0.2^ab^	391 ± 33^b^	53.7 ± 0.1^def^	0.96	0.4
CS	0	43.0 ± 0.7^de^	3.30 ± 0.2^ij^	5.5 ± 0.4^ab^	356 ± 66^bcd^	47.4 ± 0.8^hi^	0.95	0.4
HPMC L	0.5	46.8 ± 2.1^c^	3.6 ± 0.3^hi^	5.0 ± 0.8^abcd^	289 ± 58^cd^	51.9 ± 2.4^efg^	0.96	0.4
	1.0	50.0 ± 0.1^b^	4.5 ± 0.1^d^	4.1 ± 0.4^d^	395 ± 29^b^	56.1 ± 0.10^cd^	0.96	0.5
	1.5	48.6 ± 0.2^b^	4.3 ± 0.2^de^	5.3 ± 0.1^abc^	343 ± 2^bcd^	54.4 ± 0.3^cde^	0.96	0.5
	2.0	56.5 ± 0.3^a^	13.9 ± 1.8^b^	4.6 ± 0.4^bcd^	389 ± 24^bc^	73.6 ± 2.8^b^	0.95	1.7
HPMC M	0.5	45.8 ± 0.2^c^	3.7 ± 0.3^gh^	5.5 ± 0.2^ab^	329 ± 43^bcd^	50.9 ± 0.5^fg^	0.96	0.5
	1.0	45.7 ± 0.3^c^	4.3 ± 0.1^def^	5.7 ± 0.2^a^	349 ± 60^bcd^	51.3 ± 0.4^efg^	0.96	0.5
	1.5	50.3 ± 0.1^b^	5.3 ± 0.4^c^	5.4 ± 0.4^ab^	376 ± 56^bcd^	57.4 ± 0.4^cd^	0.95	0.7
	2.0	57.7 ± 0.1^a^	44.7 ± 4.7^a^	5.3 ± 0.9^abc^	597 ± 92^a^	108.4 ± 5.9^a^	0.96	4.8
HPMC H	0.5	45.4 ± 0.3^c^	3.9 ± 0.1^fg^	4.4 ± 0.2^cd^	323 ± 15^bcd^	50.7 ± 0.2^fg^	0.97	0.4
	1.0	43.7 ± 1.1^d^	4.1 ± 0.2^ef^	4.8 ± 0.4^abcd^	286 ± 31^d^	49.4 ± 1.2^gh^	0.96	0.5
	1.5	41.8 ± 0.4^ef^	4.2 ± 0.1^def^	5.0 ± 0.4^abc^	332 ± 43^bcd^	47.6 ± 0.4^hi^	0.96	0.5
	2.0	40.2 ± 0.2^f^	4.4 ± 0.2^d^	5.4 ± 0.5^ab^	362 ± 12^bcd^	46.2 ± 0.4^i^	0.96	0.6

*Data are presented as mean ± SD. Data values in a column with different superscript letters are significantly different at the p ≤ 0.05 level. WS, wheat starch; CS, corn starch.*

Analyzing the values of Burgers parameters of wheat and those obtained for CS gels with HPMC, it can be concluded that by adding from 1.0 up to 1.5% of HPMC L or 1.5% of HPMC M, similar values of J_0_, λ, μ_0,_ and J_*max*_ could be obtained. These results satisfactorily agreed with results from oscillatory tests.

## Conclusion

Water retention capacity of tested HPMC varied linearly with the average viscosimetric molecular weight (M_*v*_) of HPMC. The temperature of gel point was invariant with M_*v*_ (when substitution degree is maintained constant), but HPMC with high M_*v*_ showed an aggregation step at temperatures near (below) gel point. The HPMC addition to CS gels causes significant changes in viscoelasticity and stiffness of samples. These effects depend on the amount of polymer added and the M_*v*_ of the HPMC.

Based on the viscoelastic response and creep-recovery tests, maintaining constant solid content (30% w/w), CS gels plus HPMC with mimetic features to WS gels can be only obtained under certain circumstances. HPMC with relatively low M_*v*_ is recommendable, specifically, from 1.0 up to 1.5% (w/w) of HPMC of 27.2⋅10^3^ g/mol or 1.5% (w/w) of HPMC of 32.7⋅10^3^ g/mol must be added. Nevertheless, the use of HPMC with high M_*v*_ is not adequate for this purpose.

## Data Availability Statement

The raw data supporting the conclusions of this article will be made available by the authors, without undue reservation.

## Author Contributions

LM: methodology, data curation, and writing—original draft preparation. CR: supervision, validation, and writing—reviewing, and editing. RM: conceptualization, data curation, supervision, validation, and writing—reviewing and editing. All authors contributed to the article and approved the submitted version.

## Conflict of Interest

The authors declare that the research was conducted in the absence of any commercial or financial relationships that could be construed as a potential conflict of interest.

## Publisher’s Note

All claims expressed in this article are solely those of the authors and do not necessarily represent those of their affiliated organizations, or those of the publisher, the editors and the reviewers. Any product that may be evaluated in this article, or claim that may be made by its manufacturer, is not guaranteed or endorsed by the publisher.
